# Strategy for recruitment and factors associated with motivation and satisfaction in a randomized trial with 210 healthy volunteers without financial compensation

**DOI:** 10.1186/1471-2288-15-2

**Published:** 2015-01-05

**Authors:** Quentin Luzurier, Cédric Damm, Fabien Lion, Carine Daniel, Lucille Pellerin, Marie-Pierre Tavolacci

**Affiliations:** Rouen University Hospital, Clinical Investigation Centre - Biological Resource Centre, CIC-CRB, Inserm 1404, 1 rue de Germont, F 76031 Rouen Cedex, France; Rouen University Hospital, Emergency Care Teaching Centre, 1 rue de Germont, F 76031 Rouen Cedex, France

**Keywords:** Interventional study, Recruitment, Healthy volunteers, Motivation, Satisfaction

## Abstract

**Background:**

The aim was to describe a strategy for recruitment of healthy volunteers (HV) to a randomized trial that assessed the efficacy of different telephone techniques to assist HV in performing cardiac massage for vital emergency. Participation in the randomized trial was not financially compensated, however HV were offered emergency first-aid training. We also studied factors associated with HV motivation and satisfaction regarding participation in the trial.

**Methods:**

Strategy for recruitment of 210 HV aged 18 to 60 years was based on: (1) the updated records of all telephone number since January 2000 of HV registered in the Rouen Clinical Investigation Centre HV database, (2) a communication campaign for the general public focussing on posters and media advertisements. Data on the recruitment, socio-demographics, motivation and satisfaction of the 210 HV were collected by anonymous self-administered questionnaire.

**Results:**

Of the 210 HV included, 63.3% (n = 133) were recruited from the HV database and 36.7% (n = 77) by the communication campaign. On the one hand, the HV database enabled screening of 1315 HV, 54.8% (n = 721) of whom were reached by phone, 55.2% (n = 398) of these latter accepted to participate in the study and 10.1% of the initial screening (n = 133) were finally included. One the other hand, for the 77 HV not recruited from the HV database, word-of-mouth (56.1%) was the main means of recruitment. The male/female ratio of the 210 HV was 0.5 and mean age 43.5 years (Standard Deviation = 12.4). The main motivations given for participating in the trial were to support research (87.6%) and receive emergency first-aid training (85.7%). Overall satisfaction with the welcome process was significantly higher for older HV (46–60 years) (adjusted odds ratio (AOR): 3.44; 95% confidence interval (95% CI): 1.48-7.99), and for HV in management jobs (AOR: 4.26; 95% CI: 1.22-14.87). Satisfaction with protocol management was higher for women (AOR: 2.33; 95% CI: 1.18-4.60) and for older HV (46–60 years) (AOR: 4.76; 95% CI: 1.97-11.52).

**Conclusions:**

Recruitment of non-compensated HV required broad screening with a primary HV database alongside word-of-mouth communication which seemed more efficient than media advertising. To enhance HV recruitment to randomized trials without financial compensation it seems crucial to provide them not only with a direct interest but also to ensure their satisfaction.

## Background

Clinical trials require recruitment of patients and healthy volunteers (HV). The willingness of the general public to participate in clinical trials seems low. Ohmann et al. [1] found that only 25% of 225 visitors interviewed at a German University accepted taking part in clinical trials, with a likelihood of lower participation in surgical trials than in dental or pharmaceutical trials. Patients with cancer are often motivated to participate in order to gain possible therapeutic benefits, with pressure of growing cancer, and from study investigators and relatives [2–6]. Nevertheless, these patients also participate in helping future patients and contribute to science [3, 5, 7], especially if their prognosis is poor [7]. Patients with non-cancer diseases are largely motivated by expectation of personal benefit [8, 9] or obtaining ancillary care provided by clinical trials [10]. Altruism is also a motivation [11–17]. Moreover clinical trials also require the participation of HV, especially for phase I. The recruitment of HV is generally facilitated by providing monetary incentives [18, 19] and the main reported motivation of HV is frequently financial reward [20–26]. Kass et al. [26] investigated study participation in the USA and reported that for HV, money was a good aspect for 55% and the best aspect for 46%. But many others motivations are described by HV, such as contributing to science, helping others or personal benefits [26–29]. Few studies have been conducted in HV without financial compensation, due to funding constraints or ethical considerations. Indeed payment of HV is still discussed among volunteers: Russel et al. [27] found that a minority of Canadian HV agreed with paying research subjects. This is also discussed in the scientific community, as some authors think that money might encourage misrepresentation and the participation of disadvantaged or vulnerable persons [21, 26, 30]. Resnik [31] reviewed limits on risks in studies and reported that financial compensation should not be a justification for increased risk. The current dominant view is that only time and expenses should be compensated [32]. However without financial compensation, recruitment can be difficult. In fact, more than 50% of clinical trials require an extension because of recruitment issues and more than one-third do not achieve their original recruitment target [33], even if financial compensation is given. Moreover, to our knowledge, no study to date has reported the demographic characteristics, recruitment process, or motivation and satisfaction levels of HV who participated without financial compensation. The aim of the present study was to describe a strategy for recruitment of healthy volunteers (HV) to an interventional study without financial compensation, and to identify factors associated with the motivation and satisfaction of these HV.

## Methods

### Interventional study

The present study is ancillary to a randomized trial conducted in 2013 at the Clinical Investigation Centre (CIC) of Rouen University Hospital, France. This randomized trial had assessed the effectiveness of three telephone techniques to assist untrained volunteers in performing cardiac massage in vital emergency situations [34]. Randomization was performed by sealed envelopes and HVs were allocated into one of the three tested telephone assistances: 1- telephone assistance with the order to perform cardiac massage then hang up (control group); 2- continuous telephone assistance with an emergency regulating doctor; 3- telephone assistance with the order to perform cardiac massage then continuous guidance by a sound pacer. On the same day, the HV signed the informed consent document, was then randomized and after performed the intervention (no follow up). Then the HV performed continuum cardiac massage on a manikin during 5 minutes. The end-point of the baseline randomized trial was the effectiveness of the thoracic compression (frequency and depth). Afterwards, in order to offer a benefit and thank HVs, they received a brief emergency first-aid training session free of charge by a nurse (about 30 minutes). The randomized trial protocol was approved by the local Ethics Committee (Comité de Protection des Personnes Nord-Ouest I n° 2012/029, approved December 14, 2012). The number of subjects required was calculated for the randomized trial with a significance level of 2.5% and a power test of 80% [34]. The baseline randomized trial required recruitment of 210 non-financially compensated HV and was conducted for 7 days between September and November 2013 at the CIC. The CIC managed recruitment of the HVs required, and assessed the strategy for recruitment as well as the motivation and satisfaction of the HVs. The present study reports the results of this assessment. The CIC is authorised to conduct interventional studies with HV.

### Eligibility criteria

Only healthy volunteers age from 18 to 60 years were eligible for inclusion. Non-inclusion criteria were: current employment or volunteering as rescuers (fire fighters, ambulance attendants or first aid volunteers), emergency first-aid training within 12 months of the study or rescuer, physical or mental disability, medical contraindication to physical exertion, low proficiency in French language, pregnancy or breastfeeding, or legal protection (curatorship or guardianship).

### Recruitment

The first stage in the recruitment process of HV was the use of the Rouen CIC database: “Logic CIC”, (Oriam® eTM@V3). Logic CIC is a software programme which is specially designed by the French National Institute of Health and Medical Research (INSERM) to assist CICs in France in managing their own HV database. Healthy volunteers wishing to participate in medical research protocols are registered in this database with their demographic characteristics (last name, first name, gender, date of birth, place of birth, profession), life-style characteristics (contraception, smoking, alcoholism) and their contacts (address, telephone numbers). Our registration of HV began in January 2000 and included an overall 1500 HV at the moment of extraction. The database is updated when HVs self report any changes and when participating in a study. The database was declared to the National Commission on Informatics and Liberty (Commission Nationale Informatique et Libertés N° 1383369). Logic CIC allows selection of HVs according to their demographic and life-style characteristics, and contacts. Recruitment for the baseline randomized trial was based on extraction of the mobile and land line telephone numbers of all HV aged 18 to 60 years registered in Logic CIC on August 27, 2013. We attempted to contact by telephone all HV extracted from Logic CIC and proposed participation in the study to those HV successfully contacted. The randomized trial was presented as medical research on emergency first aid, with an emergency first-aid training session. If participation in the study was declined, the reason for refusal was requested by an open-ended question, the answer to which was retrospectively categorized as “HV not available”, “no financial compensation”, “other” or “reason not given”. If the HV agreed to participate and was available during the proposed period (appointments between 8:30 and 16:30), non-inclusion criteria were checked by telephone, starting with “emergency first-aid training within 12 months of the study or rescuer”, then “physical disability”, then “medical contraindication to physical exertion”. As soon as one non-inclusion criterion was reached, no further questions were asked. The criteria “mental disability” and “legal protection” were not asked by telephone. The criterion “low proficiency in French language” was evaluated, and some pregnancies were spontaneously reported by HV. In absence of non-inclusion criteria, an appointment was given for the inclusion visit at the CIC. Other methods of communication were used to recruit the 210 HV: posters (in Rouen: university hospital, faculty of medicine, faculty of law, local businesses), a message on Rouen University Hospital’s website, in newspapers (local daily newspaper), on radio and on social networks (Facebook, Twitter).

### Data collection

HV were included in the study after interview at the CIC, verification of selection criteria and signature of consent. They then participated in the trial on the same day (one visit: information, inclusion, intervention). After the intervention (cardiac massage), the HV completed an anonymous self-administered questionnaire. Demographics were collected on gender, age and socio-professional category, classified as follows: without professional activity (unemployed, inactive or retired), student, farmer, blue-collar worker, employee, middle-level activity (mid-level profession, shop keeper, craftsman or business owner) and manager/intellectual profession. Information’s source of the study’s conduct were collected: word-of-mouth, posters, Rouen University Hospital website, newspapers, radio, social networks, and others (several answers possible). The HV not registered in Logic CIC database were those recruited by these other communication strategies. Participation in other clinical trials and motivation to participate in the present study were: to support research, learn about emergency first-aid, update knowledge of emergency first-aid, curiosity, interest in medical research results, protocol without risk (no needle, no drugs etc.), and other choices (several answers possible). Prospects for HV participation were also collected: participation in a new medical research study, recommendations for relatives to participate in a new medical research study, interest in possibility of consulting the research protocols proposed by the CIC on the Internet and to register directly online (5-item Likert scale: “strongly disagree” to “completely agree”). Some data were not reported by HV (gender, age, socio-professional category, motivation, satisfaction, participation outlook). Thus we have indicated the maximal number of respondents to every question.

### Satisfaction scores

Satisfaction on the overall welcome at the CIC was measured by 5-item Likert scale (0: “unsatisfied” to 4: “very satisfied”) for each of the following items: directions given by the staff for finding the CIC, ease in finding the CIC, rapidity of greeting on arrival at the CIC, cleanliness of the premises. An overall satisfaction score of the welcome was obtained by summing the 4 items (score from 0 to 16). Satisfaction to the protocol management was also measured by a 5-item Likert scale (0: “unsatisfied” to 4: “very satisfied”) for each of the following items: explanation of the research interest by the physician, explanation of protocol organisation by the staff, time for reflection suggested by the physician between information form and consent signature, physician’s explanation of benefits and risks of the study, confidentiality during the study. An overall satisfaction score of the protocol management was obtained by summing the 5 items (score from 0 to 20).

### Statistical analysis

Age was categorized into three groups for analyses (18–30, 31–45 and 46–60 years). The age cut-offs were chosen to form approximately equal periods of time (about 15 years for each age group). In our study, the median was high for the satisfaction scores related to overall welcome and protocol management. Satisfaction scores were dichotomized into two classes according to the median: not fully satisfied HV (score below or equal to the median) and fully satisfied HV (score above the median). Percentages were used to describe HV responses. There were some missing data (i.e. no response to items in the self-administered questionnaire by the 210 HV): 7 on gender, 8 on age, 10 on socio-professional category, 4 on overall satisfaction with the welcome process and 10 on satisfaction with protocol management. Chi-square tests were conducted to determine differences in categorical data and chi-square tests for linear trend (extended Mantel-Haenszel) to analyse the variables according to age. We performed two multivariate analyses to identify factors associated with full satisfaction using logistic regression for each of the two satisfaction scores (Overall welcome satisfaction and Protocol management satisfaction) as dependant variables (two classes: not fully satisfied or fully satisfied HV). Factors with a p value lower than 0.30 (gender, age, socio-professional category and registration status in Logic CIC database) were included in the multivariate analysis and a p value lower than 0.05 was considered to be significant. Statistical analyses were performed using StatCalc Epi Info® 7.

## Results

### Recruitment process

**Figure 1:**
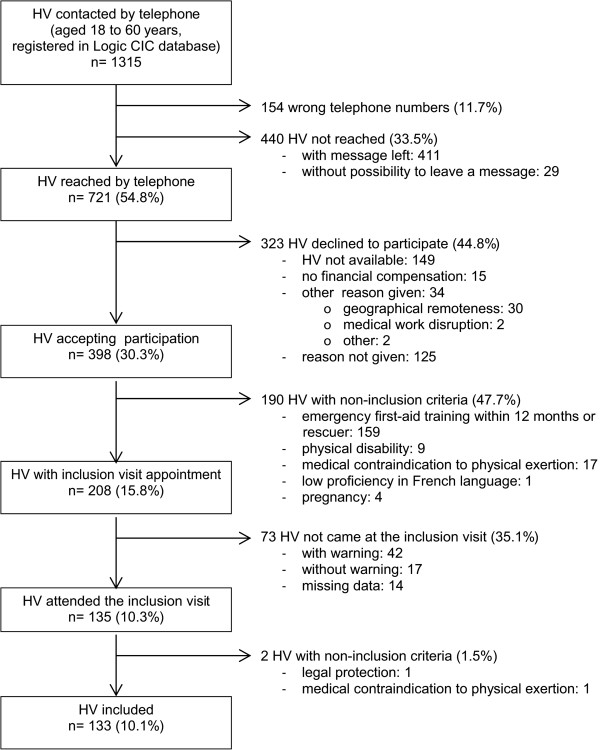
**Flow chart of healthy volunteer recruitment, based on Logic CIC database.** HV: healthy volunteers. CIC: Clinical Investigation Centre.

The telephone numbers of 1315 HV aged 18 to 60 years were extracted from Logic CIC database. The male/female ratio was 1.3. The number of HV at each stage of recruitment is shown in the flow chart (Figure 1). Of the 1315 HV, 133 were finally recruited for the randomized trial (10.1%, 95% CI: 8.5 – 11.7). Table 1 shows the demographics of HV registered in Logic CIC database at the three main stages of recruitment. Greater age of HV was associated with easier contact by telephone (358/776, 46.1% of under 30 year old HV; 112/180, 62.2% of 31 to 45 year old HV and 250/354, 70.6% of 46 to 60 year old HV; p < 0.001). Overall, 133 of the 210 HV (63.3%) included in the intervention study were recruited by this process. Five HV did not report their registration in the anonymous self-administered questionnaire. Of the 82 HV who had declared they were not registered in Logic CIC database, the main source of information was word-of-mouth (46/82 HV, 56.1%). Sources of information according to gender and age are reported in Table 2. Word-of-mouth was most frequently reported by younger HV (22/29, 75.9% of under 30 year old HV; 9/23, 39.1% of 31 to 45 year old HV and 13/27, 48.2% of 46 to 60 year old HV; p = 0.02).

**Table 1:** **Demographics of healthy volunteers registered in Logic CIC database at the 3 main stages of recruitment (N = 1315)**

	Overall (%)	Gender (%)			Age (%)			
		Men	Women	p	≤30	31 to 45	46 to 60	p ^†^
HV reached (among HV called, n = 1315)	54.8	56.3	52.9	0.23	46.1	62.2	70.6	<0.001
HV accepting participation* (among HV reached, n = 721)	55.2	56.0	54.0	0.59	51.4	59.8	58.8	0.07
HV attended the inclusion visit (among HV pre-included, n = 208)	64.9	64.9	64.9	0.99	65.2	58.7	67.7	0.75

*Request for participation before seeking non-inclusion criteria.

^†^Chi square for linear trend (extended Mantel-Haenszel).

HV: healthy volunteers.

**Table 2:** **Sources of information on the study for healthy volunteers not registered in Logic CIC database, several answers possible (N = 82)**

	Overall (% (n))	Gender (%)			Age (%)			
	N = 82	Men n = 25*	Women n = 54*	p	≤30 n = 29*	31 to 45 n = 23*	46 to 60 n = 27*	p ^†^
Word-of-mouth	56.1 (46)	52.0	57.4	0.65	75.9	39.1	48.2	0.02
Posters	15.9 (13)	12.0	16.7	0.59	20.7	13.0	11.1	0.24
Newspapers	13.4 (11)	24.0	9.3	0.08	6.9	17.4	18.5	0.29
Hospital website	11.0 (9)	12.0	11.1	0.91	10.3	8.7	14.8	0.76
Social networks	4.9 (4)	0.0	5.6	0.23	6.9	4.4	0.0	0.09
Radio	2.4 (2)	4.0	1.9	0.57	0.0	8.7	0.0	0.70
Other	17.1 (14)	8.0	22.2	-	10.3	21.7	22.2	-

*Missing data on gender and age for 3 healthy volunteers.

^†^Chi square for linear trend (extended Mantel-Haenszel).

### Description of HV included

The male/female ratio of the 210 HV included was 0.5 and mean age was 43.5 years (Standard Deviation = 12.4). Age groups were: under 30 years (34.7%), 31–45 years (25.2%) and 46–60 years (40.1%). HV were: without professional activity (12.5%), students (15.5%), blue-collar workers (5.0%), employees (40.5%), mid-level professions (13.5%) and managers/intellectual professions (13.5%). There were no farmers. Ninety-seven HV (46.2%) had already participated in a medical research study.

### Motivation of HV included

The motivations of the HV participating in the study are presented in Table 3. The main motivation was supporting medical research, reported by 87.6% (184/210) of HV. Emergency first-aid training (“learning about emergency first-aid” or “updating knowledge on emergency first-aid”) was reported by 85.7% (180/210) of the HV. Supporting medical research was reported significantly more by women (54/68, 79.4% of men and 123/135, 91.1% of women; p = 0.02) and by Logic CIC database registered HV (67/82, 81.7% of unregistered HV and 117/128, 91.4% of registered HV; p = 0.04). Interest in medical research results was more frequently reported by older HV (18/70, 25.7% of under 30 year old HV; 14/51, 27.5% of 31 to 45 year old HV and 40/81, 49.4% of 46 to 60 year old HV; p = 0.003) and curiosity by younger HV (31/70, 44.3% of under 30 year old HV; 14/51, 27.5% of 31 to 45 year old HV and 21/81, 25.9% of 46 to 60 year old HV; p = 0.01).

**Table 3:** **Motivation, satisfaction and participation outlook of healthy volunteers (N = 210)**

	Overall	Gender (%)			Age (%)				CIC computer file (%)		
	(%) N = 210	Men n = 68	Women n = 135	p	≤30 n = 70	31 to 45 n = 51	46 to 60 n = 81	p ^‡^	Unregistered n = 82	Registered n = 128	p
Motivation (several answers possible)											
Supporting research	87.6	79.4	91.1	0.02	84.3	80.4	93.8	0.09	81.7	91.4	0.04
Learning about emergency first-aid	58.1	58.8	57.0	0.81	55.7	66.7	53.1	0.64	63.4	54.7	0.21
Updating knowledge of emergency first-aid	51.9	54.4	50.4	0.59	54.3	37.3	58.0	0.64	45.1	56.3	0.12
Interest in medical research results	35.2	35.3	35.6	0.97	25.7	27.5	49.4	0.003	31.7	37.5	0.39
Curiosity	32.9	29.4	34.1	0.50	44.3	27.5	25.9	0.01	30.5	34.4	0.56
Protocol without risk	11.4	5.9	14.8	0.06	11.4	9.8	13.6	0.76	14.6	9.4	0.24
Other*	2.4	2.9	2.2	-	0.0	3.9	3.7	-	2.4	2.3	-
Satisfaction											
Overall welcome (overall score > 15)	49.0	40.9	53.4	0.10	36.2	40.8	65.0	0.001	44.3	52.0	0.28
Protocol management (overall score > 19)	40.0	29.4	45.5	0.03	25.7	34.7	55.0	<0.001	35.4	43.0	0.29
Participation outlook											
Participation in a new research study	98.5	98.5	98.5	0.99	97.1	100.0	98.8	0.66	96.2	100.0	0.03
Recommendations for relatives to participate	99.0	100.0	98.5	0.31	98.6	98.0	100.0	0.62	100.0	98.4	0.26

*Others reasons: civic mindedness, altruism, to be recruited for a future trial.

^‡^Chi square for linear trend (extended Mantel-Haenszel).

### Satisfaction of HV included

**Figure 2:**
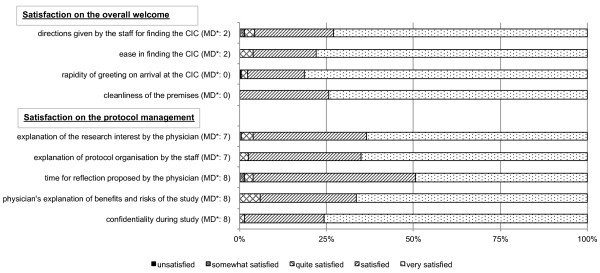
**Answers for each satisfaction item of self-administered questionnaire (N = 210).** *MD: number of missing data (i.e. HV who did not respond to a satisfaction item). CIC: Clinical Investigation Centre.

Reponses for each satisfaction item on the self-administered questionnaire are shown in Figure 2. Median, minimum, and maximum satisfaction scores for the overall welcome were 15, 10 and 16 (out of 16) respectively. This satisfaction score differed significantly according to age (p = 0.001) but not to gender or registration in Logic CIC database (Table 3). After multivariate analysis (Table 4), HV aged 46–60 years and managers/intellectual professions were significantly more satisfied with the overall welcome, with respectively AOR: 3.44, 95% CI [1.48 - 7.99] and AOR: 4.26, 95% CI [1.22 - 14.87]. Median, minimum, and maximum satisfaction scores for protocol management were 19, 11 and 20 (ouf of 20) respectively. This satisfaction score differed significantly according to gender (p = 0.03) and age (p < 0.001) (Table 3). After multivariate analysis (Table 4), women and HV aged 46–60 years were significantly more satisfied with the protocol management, with respectively AOR: 2.33, 95% CI [1.18 - 4.60] and AOR: 4.76, 95% CI [1.97 - 11.52].

**Table 4:** **Overall welcome and protocol management satisfaction of healthy volunteers, multivariate analysis (N = 210)**

	Overall welcome satisfaction			Protocol management satisfaction		
	AOR*	95% CI	p	AOR*	95% CI	p
Gender						
Male	1.00			1.00		
Female	1.74	0.90 - 3.39	0.10	2.33	1.18 - 4.60	0.01
Age						
≤30	1.00			1.00		
31 to 45	1.15	0.48 - 2.80	0.75	2.10	0.82 - 5.37	0.12
46 to 60	3.44	1.48 - 7.99	<0.01	4.76	1.97 - 11.52	<0.01
Socio-professional category						
Without professional activity	1.00			1.00		
Student	1.39	0.38 - 5.04	0.62	1.92	0.50 - 7.33	0.34
Blue-collar worker	0.37	0.06 - 2.31	0.29	0.92	0.17 - 4.98	0.92
Employee	1.55	0.58 - 4.15	0.39	1.37	0.50 - 3.73	0.54
Mid-level activity	1.73	0.51 - 5.88	0.38	1.17	0.34 - 3.99	0.81
Managers/intellectual profession	4.26	1.22 - 14.87	0.02	1.47	0.45 - 4.83	0.53
Logic CIC database						
Unregistered^†^	1.00			1.00		
Registered	1.35	0.71 - 2.56	0.36	1.33	0.70 - 2.53	0.39

Missing data: 15 healthy volunteers for overall satisfaction with welcome process; 14 healthy volunteers for satisfaction with protocol management.

*Adjusted Odds Ratio to others factors listed in the table, logistic regression.

^†^Healthy volunteers unregistered in Logic CIC database were those recruited by the other communication strategies.

## Discussion

### Recruitment process of HV

The HV database of the CIC of Rouen (Logic CIC) was large and allowed extraction of telephone numbers of 1315 HV aged 18 to 60 years. The rate of recruitment by this HV database was 10.1% and represented 63.3% of the total sample required demonstrating the value of such a database for recruitment of HV. Greater age of HV was associated with easier contact by telephone. An assumption may be that young HV more likely had a mobile telephone, providing more difficulties to reach them than with a land line, because of number changes or technical problems such as regional coverage. Pitrou et al. [35] found that French people using only mobile telephones were under 40 years and that study participants contacted on their mobile telephone appeared to be less easily reachable than by land line. This possible confounder is minimized because the telephone numbers provided by HV to register on Logic CIC database were the numbers which were the easiest reachable.

Furthermore in our study, the main reason given by HV who had been successfully contacted for their refusal to participate was lack of availability during the proposed time slots. Logistical issues appeared as a major obstacle in recruitment, such as inability to take extra time off work for research appointments [17]. Moreover the appointment reliability of HV was also problematic: in our study, 35.1% of HV with an appointment did not attend and some without advance warning. Thus the study which was initially planned to last 5 days, finally required 7 days.

Word-of-mouth was the main source of information for HV not recruited by database, highlighting the importance of satisfied HW to encourage their relatives to participate in the current study and so enhancing of the recruitment. According to the literature, the sources of information in our study are consistent. A study performed in 2008–2009 in South-Korea [25] with 151 HV reported word-of-mouth as the widest source of information, followed by posters or booklets in a hospital. In order to improve the recruitment of volunteers (healthy or patients), other strategies are proposed in the literature [19, 33, 36, 37]: telephone reminders to non-respondents, use of opt-out rather than opt-in procedures for contacting potential participants, newsletters/mailshots/flyers, regular visits/telephone calls, posters/information leaflets in clinics/wards/notes, or change of inclusion criteria/protocol amendment. In our study, posters and advertisements in the press and on the hospital website seemed to be moderately effective while radio and social networks did not seem very useful for recruitment. Then means and time spent for the realization of these materials should not be a priority in a strategy for recruitment.

### Motivations of HV to participate in a non-financially compensated trial

The non-inclusion criterion of emergency first-aid training within the previous 12 months or rescuer status could partly explained the low rate of participations of students (15.5%) because students had often received first-aid training. Moreover, this low participation could be due to absence of financial compensation, as Van Gelderen et al. [38] reported that younger volunteers (18–30 years) were more likely to cite money as a reason for participation than older volunteers. Furthermore the most represented socio-professional category was employees (40.5%) Other socio-demographic factors could influenced the participation of HV in clinical trials: volunteers in a higher socioeconomic class are more likely to participate in Phase I studies [24] and Caucasians with a higher education level are more motivated by financial reward [26]. One special feature of our study was absence of any financial compensation for HV. Emergency first-aid training (“learning about emergency first-aid” or “updating knowledge of emergency first-aid”) was reported to be a major motivation by most HV (85.7%). Training presented a personal benefit, probably encouraging HV participation despite absence of financial compensation, which certainly facilitated recruitment. Moreover refusal to participate due to absence of financial compensation was little reported in our study (4.6% of the 323 HV contacted by telephone who refused to participate) but under-reporting of this reason is likely, especially among those who gave no reason for refusal (38.7%). A review of the literature [28] showed that although financial reward is the primary motivation for HV participating in clinical trials, other motivations are reported (contributing to science or the health of others, accessing ancillary healthcare benefits, scientific interest or interest in the goals of the study, meeting people and curiosity). In our study, motivation “protocol without risk” was little reported, appearing to be a relatively low decisive criterion for participation in the trial when the risk was low. The literature shows that volunteers considered the risk when making decisions regarding participation [39, 40] and that the risk of the study was the ultimate deciding factor for the volunteers [41].

### Satisfaction of HV

In our study, satisfaction for the overall welcome and for the protocol management was very high. A survey conducted in 2004–2005 at the CIC of Grenoble, France [42] also reported high satisfaction, but did not differentiate gender of participants, whereas in our study women were more satisfied than men regarding protocol management. Satisfaction is important as it improves future recruitment by the positive effect it may have on recruiting repeat volunteers, and even on recruitment of naive volunteers, especially by word-of-mouth. Indeed volunteers consider staff behaviour, their relationship with other volunteers, and other aspects of the study environment to have a large impact on their well-being while participating in the study [26, 43].

### Study limitations

Our study has several limitations. First, telephone reachability could be a recruitment bias, indeed successful telephone contact was associated with age: older HVs were significantly more reachable than younger HVs (70.6% vs 46.1%). Second, self-reporting of measures presented a risk of classification bias. Third, satisfaction was very high and therefore analysis was a comparison of fully satisfied HV and not fully satisfied HV. This low variability criterion could result in non-identification of significant association. The interpretation of satisfaction according to socio-demographic characteristics must remain cautious, other unstudied factors could explain the difference in HV satisfaction.

## Conclusions

The main strength of our study was the large sample size of HV recruited, despite absence of financial compensation. The recruitment of HV aged 18 to 60 years without financial compensation required broad screening with an essential updated HV database. Word-of-mouth appeared to be a more efficient source of information on the study than media advertising. One of the main motivations for participating was to benefit from a brief emergency first-aid training session at the end of the trial. Thus recruitment strategy of HV in randomized studies without financial compensation should be enhanced by providing direct interest to HV and ensuring HV satisfaction.
